# Apresentação Semelhante a um Infarto de Miocardite Fulminante Fatal Biventricular em um Paciente com Timoma

**DOI:** 10.36660/abc.20230868

**Published:** 2024-09-06

**Authors:** Fabio Maramao, Fabio Stefano Maramao, Laura Sanchez Monteso, Mirella Marino

**Affiliations:** 1 IRCCS Regina Elena National Cancer Institute Roma Itália IRCCS Regina Elena National Cancer Institute, Roma – Itália; 2 Campus Bio-Medico University Hospital Foundation Roma Itália Campus Bio-Medico University Hospital Foundation, Roma – Itália; 3 University of Rome Tor Vergata Polyclinic Tor Vergata Roma Itália University of Rome Tor Vergata, Polyclinic Tor Vergata, Roma – Itália

**Keywords:** Miocardite, Timoma, Infarto do Miocárdio

## Introdução

Timomas são tumores epiteliais raros do mediastino frequentemente associados a doenças paraneoplásicas,^1^ a miocardite é uma complicação rara em pacientes afetados por timoma.^2^ Polimiosite e miocardite associadas ao timoma são condições excepcionalmente raras e geralmente acompanhadas de miastenia gravis (MG).^3^

A miocardite, uma doença inflamatória do músculo cardíaco, pode resultar de uma ampla variedade de causas infecciosas, tóxicas e autoimunes. Embora o curso da doença seja geralmente autolimitado, a miocardite aguda e não fulminante pode evoluir para miocardite fulminante. A miocardite não fulminante tem apresentação tipicamente insidiosa e pode passar inadvertida à medida que progride para a fase crônica da doença. A definição de miocardite fulminante evoluiu desde sua descrição original em 1991.^4^ Na sua forma fulminante, a miocardite é uma doença com distúrbios hemodinâmicos e arritmias ventriculares devido a um processo inflamatório grave que requer suporte da função da bomba cardíaca e/ou tratamento urgente de arritmias graves.^5^ O reconhecimento precoce e o manejo agressivo são essenciais para um resultado favorável.^6^ A miocardite aguda pode ocasionalmente ter uma apresentação semelhante a um infarto, com dor torácica, elevação do segmento ST no eletrocardiograma e níveis elevados de troponina.^7^

Descrevemos um caso singular de miocardite fulminante fatal biventricular com apresentação semelhante a um infarto, ocorrendo em um paciente com timoma.

## Descrição do caso

Em 20 de fevereiro um paciente de 52 anos deu entrada em nosso Instituto com dor torácica atípica nas últimas duas semanas.

O paciente era fumante (15 cigarros por dia há cerca de 30 anos) e bebedor de vinho (2 a 3 taças de vinho por dia). Nefrolitíase prévia à direita. O paciente relatava história familiar de infarto agudo do miocárdio; na sua história clínica estavam ausentes doenças relevantes.

A RX do tórax e a tomografia computadorizada torácica (TC) revelaram uma massa mediastinal anterior de 11 cm de diâmetro.

**Figura 1:**
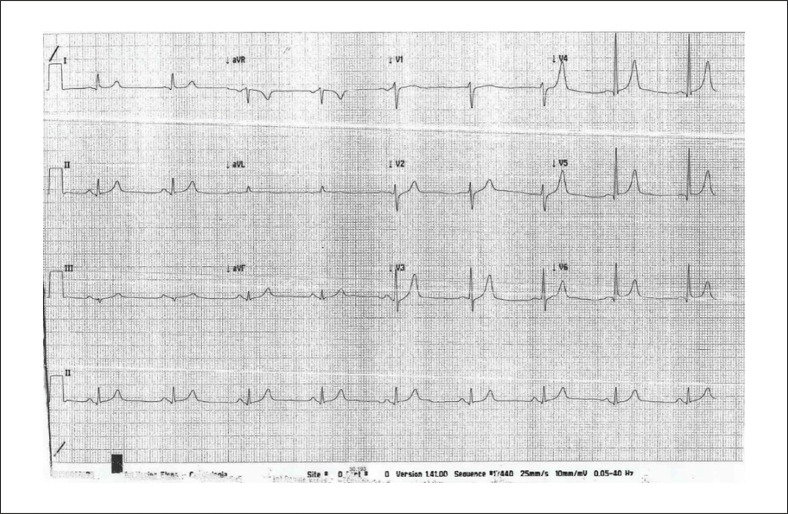
– ECG da admissão: Bradicardia sinusal, frequência cardíaca 53 batimentos/minuto; eletrocardiograma normal.

Na admissão o paciente apresentava boa compensação cardioculatória, pressão arterial normal (110/70 mmHg), frequência cardíaca de 53 batimentos/minuto; o eletrocardiograma (ECG) e o ecocardiograma foram normais (Figuras 1 e 2), principalmente a espessura do septo interventricular era normal e não havia evidência de espessamento da parede do ventrículo direito.

**Figura 2:**
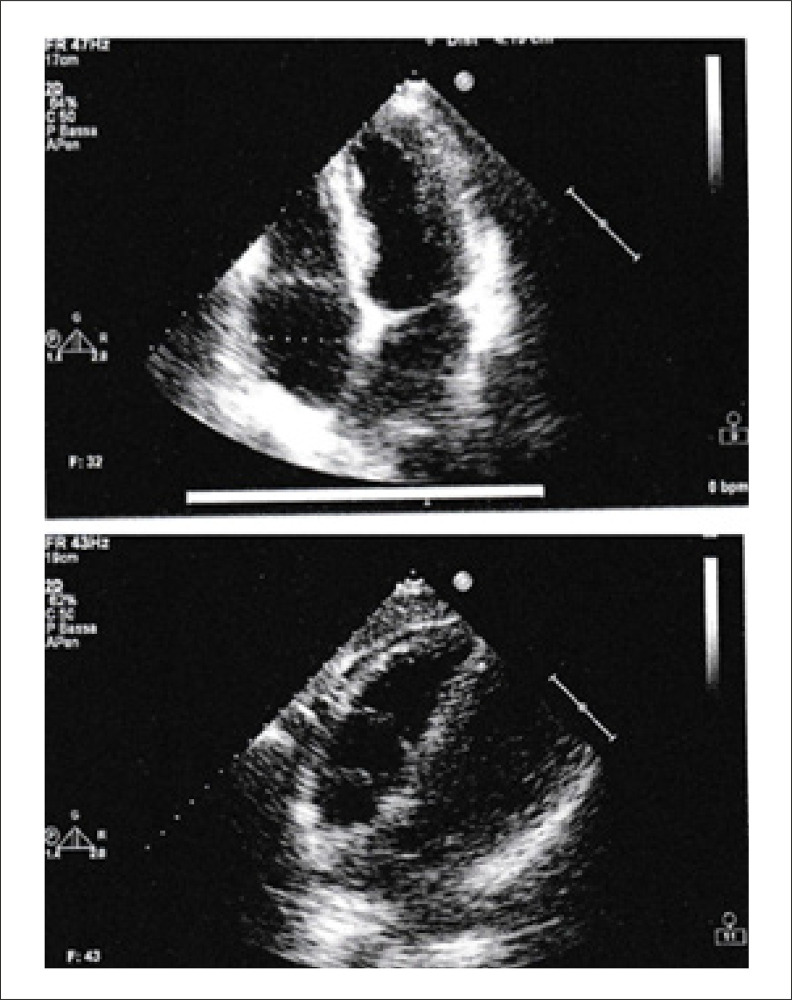
– Ecocardiograma na admissão: a espessura do septo interventricular era normal e não havia evidência de espessamento da parede do ventrículo direito.

Foram realizadas mediastinotomia anterior direita, pleurotomia e biópsia mediastinal.

**Figura 3:**
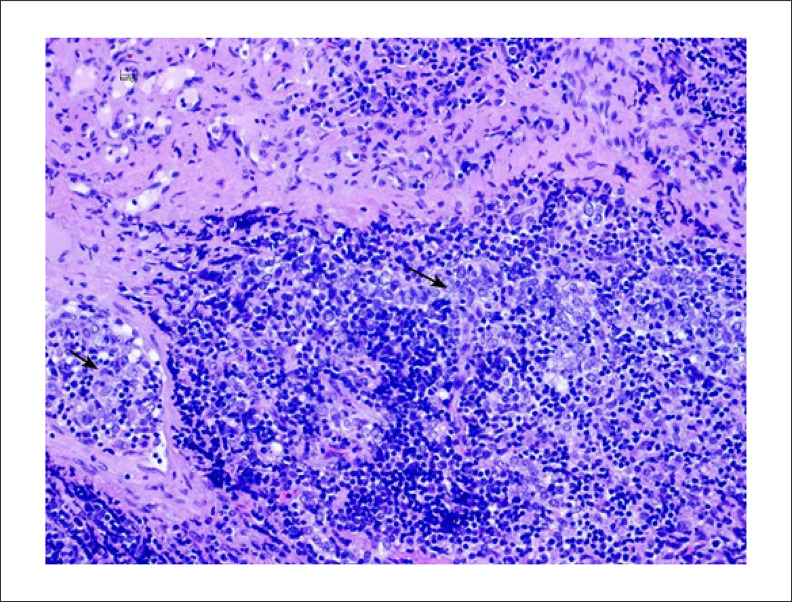
– Coloração hematoxilina-eosina (20x) da biópsia do tumor mediastinal: redes e grupos de células epiteliais (setas pretas) são vistos entre infiltração de células linfoides T.

O diagnóstico da biópsia foi timoma, provavelmente B2 (de acordo com a classificação da OMS 2004). Foi encontrada uma mistura de células epiteliais e de linfócitos (Figura 3).

Duas populações de células T foram encontradas por imunocitofluorimetria no sangue periférico e no aspirado de medula óssea: no entanto, a imunofenotipagem mostrou uma linfocitose policlonal reativa de células T. A consulta hematológica, portanto, excluiu uma doença linfoproliferativa clonal de células T.

O 14 de março de 20 paciente foi submetido a quimioterapia neoadjuvante para timoma invasivo: Adriblastina 110 mg e Cisplatina 140 mg. 21 dias após a quimioterapia, a tomografia computadorizada de tórax mostrou redução da massa mediastinal.

O 19 maio de 20 surgimento de dores nas pernas, diminuindo com o movimento, agravamento da dor no peito e aumento das transaminases. O eletrocardiograma apresentava ritmo sinusal, frequência cardíaca de 89 batimentos/minuto, bloqueio de ramo direito e bloqueio fascicular anterior esquerdo.

**Figura 4:**
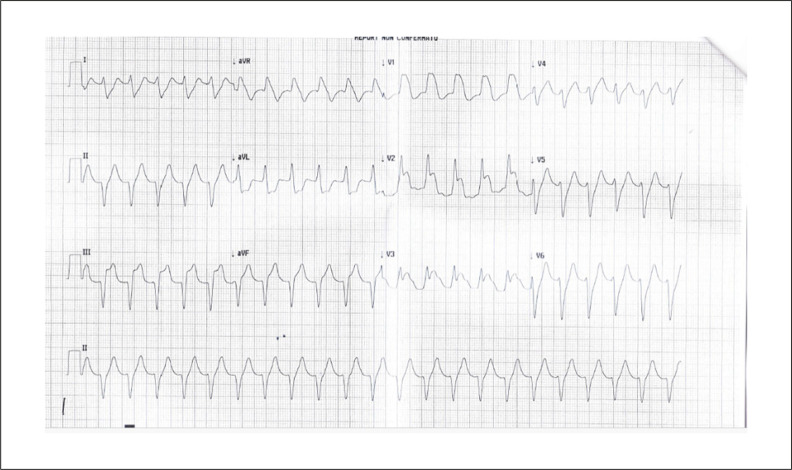
– ECG 21 maio 20: taquicardia com complexo QRS largo, frequência cardíaca de 133 batimentos/minuto, bloqueio completo de ramo direito, supradesnivelamento do segmento ST em V1-V3 simulando infarto agudo do miocárdio; ECG anormal.

Dois dias depois, devido à dor torácica persistente e ao aparecimento de sinais e sintomas de insuficiência cardíaca, foi realizada avaliação cardiológica de urgência: a pressão arterial era indetectável, o pulso carotídeo detectável e o eletrocardiograma mostrava taquicardia de complexo QRS largo, frequência cardíaca de 133 batimentos/minuto, bloqueio completo de ramo direito, supradesnivelamento do segmento ST em V1-V3 simulando infarto agudo do miocárdio (Figura 4).

**Figura 5:**
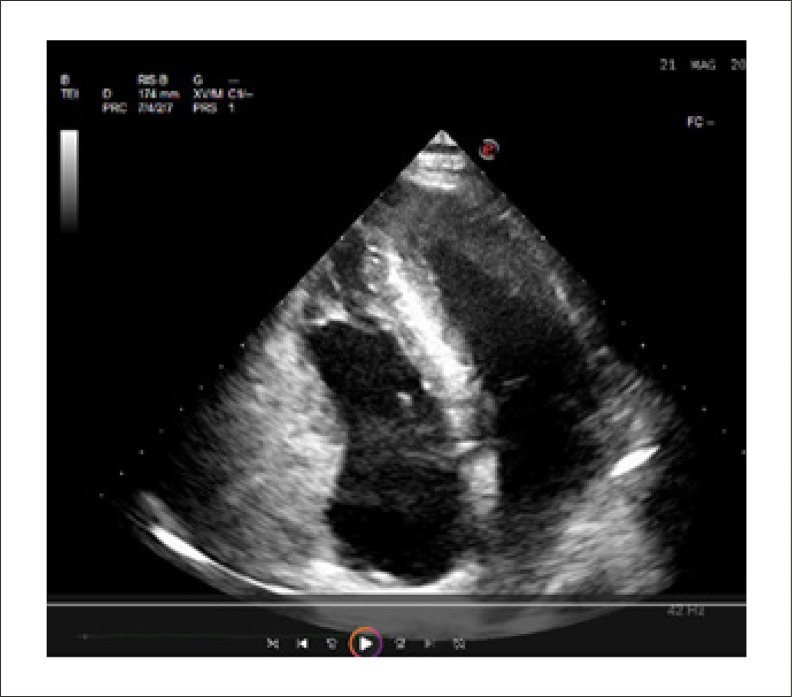
– Ecocardiograma 21 maio 20**: aumento da espessura da parede ventricular direita e esquerda e aumento da espessura do septo interventricular.

O ecocardiograma mostrou redução moderada da fração de ejeção do ventrículo esquerdo (40%), redução (qualitativa) da fração de ejeção do ventrículo direito, discinesia do septo interventricular, aumento da espessura da parede do ventrículo direito e esquerdo e aumento da espessura do septo interventricular (14 mm) (Figura 5).

Os marcadores bioquímicos de lesão miocárdica estavam aumentados: troponina T de alta sensibilidade (hs-cTnT) > 10.000 mg/L e massa MB de creatinoquinase > 600 mg/ml. Foram administrados ácido acetilsalicílico, clopidogrel, morfina, oxigênio, corticoide, infusão líquida e heparina de baixo peso molecular.

## Resultado

Apesar da terapia administrada, a insuficiência cardíaca piorou rapidamente e o paciente morreu após algumas horas.

## Discussão

**Figura 6:**
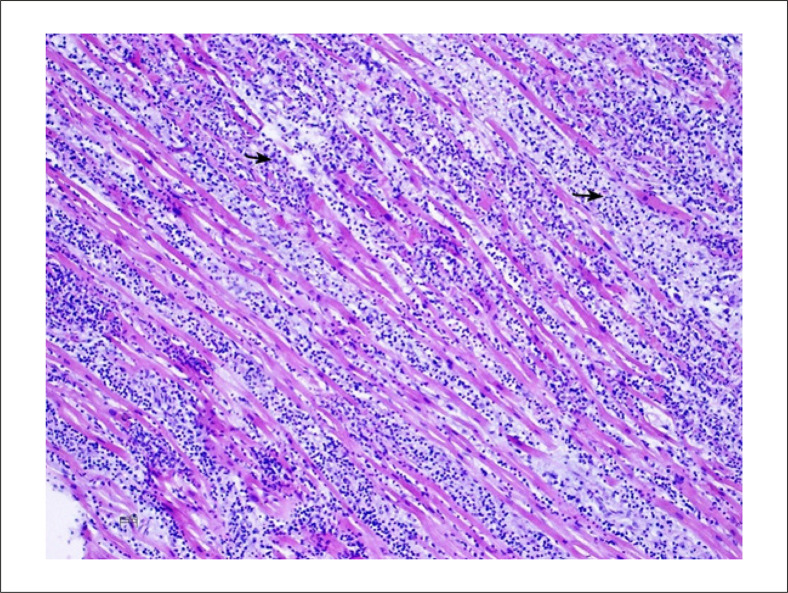
– Miocárdio na autópsia: infiltração linfo-histiocítica (setas pretas) destruindo fibras musculares cardíacas (Hematoxilina-Eosina, 10x).

Na autópsia foi encontrado amplo timoma necrótico residual, infiltrando o pulmão direito. A autópsia mostrou miocardite biventricular resultando em dano isquêmico, mais evidente no ventrículo direito, com dano miocárdico secundário à infiltração linfoide T histiocítica e policlonal reativa (Figura 6).

**Figura 7:**
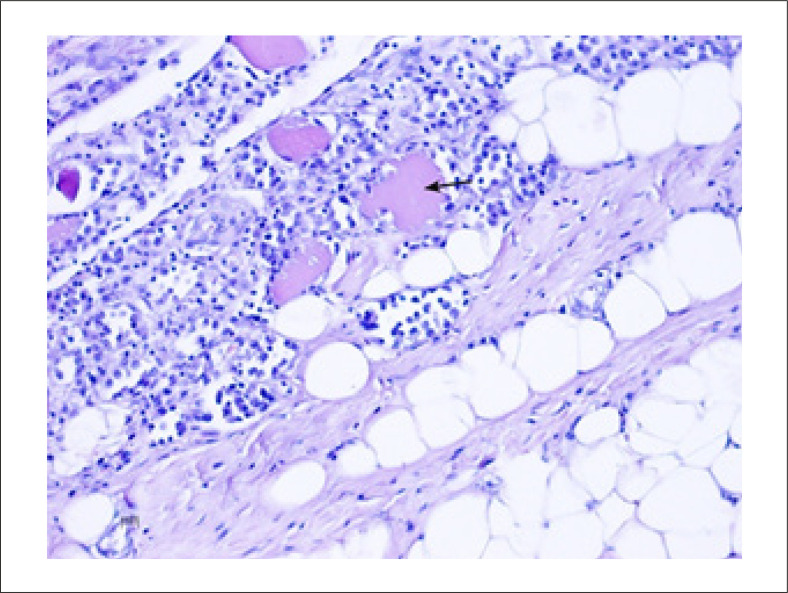
– Músculos intercostais (parede torácica anterior) na autópsia: infiltração linfo-histiocítica destruindo fibras musculares esqueléticas (seta preta) (Hematoxilina-Eosina, 20x)

O mesmo tipo de infiltração linfo-histiocítica foi encontrado entre as fibras musculares da parede torácica peristernal (Figura 7).

Houve hipertrofia biventricular reativa secundária, dano isquêmico miocárdico e muscular esquelético; o dano isquêmico miocárdico, detectado na autópsia, poderia ser responsável pela apresentação eletrocardiográfica com elevação do segmento ST (apresentação semelhante a infarto de miocardite).

A avaliação molecular realizada na biópsia e no material autóptico foi negativa para identificação de rearranjos clonais do gene da cadeia gama receptora de células T.

A miocardite pode estar relacionada com vários agentes/mecanismos^8^ e, de entre estes, tanto com fenómenos auto-imunes como com lesões provocadas por fármacos, também por antraciclinas.

A apresentação clínica com sintomas de miocardite e miosite nos permitiu excluir a miocardite induzida por antraciclinas; de fato, a miosite não está descrita como uma complicação da terapêutica com antraciclinas. Além disso, os sinais morfológicos de lesão miocárdica induzida por antraciclinas são alterações degenerativas e necróticas nos miócitos, vacuolização, lesão mitocondrial e infiltrado inflamatório focal limitado.^9^ Em vez disso, no nosso caso, foi encontrado um infiltrado inflamatório grave e difuso nas células musculares estriadas do coração e dos músculos esqueléticos.

Diagnóstico final

Miocardite difusa resultando em dano isquêmico e miosite difusa (com infiltração de células T policlonais) em paciente com timoma invasivo em tratamento. Edema pulmonar e choque cardiogênico.

## Conclusão

Dor torácica e dispneia são sintomas frequentes em pacientes com timoma, mas podem ocorrer também na miocardite.^8^ A polimiosite e a miocardite associadas ao timoma são condições excepcionalmente raras e geralmente acompanhadas de MG.

Portanto, em pacientes com timoma, é sempre necessário considerar a ocorrência de miocardite para estabelecer terapia precoce, adequada e agressiva: na verdade, a inflamação do miocárdio também pode se desenvolver de forma repentina e grave, resultando em necrose de miócitos, edema e choque cardiogênico, específico sinais e sintomas de miocardite fulminante.

Além disso, o paciente com miocardite aguda pode apresentar dor torácica, elevação do segmento ST no eletrocardiograma e níveis elevados de troponina. Portanto, deve ser feito um diagnóstico diferencial com infarto agudo do miocárdio.
