# Supplementary material for: Apresentação Semelhante a um Infarto de Miocardite Fulminante Fatal Biventricular em um Paciente com Timoma
Source: Arq Bras Cardiol. 2024 Sep 6;121(8):e20230868. [Article in Portuguese] doi: 10.36660/abc.20230868 (PMC11495800; doi:10.36660/abc.20230868)
Supplement: Translation

# Translation: Biventricular Fatal Fulminant Myocarditis Infarct-Like Presentation in a Patient with Thymoma

**DOI:** 10.36660/abc.20230868i

**Authors:** Fabio Maramao, Fabio Stefano Maramao, Laura Sanchez Monteso, Mirella Marino

**Affiliations:** 1 Italy IRCCS Regina Elena National Cancer Institute, Roma – Italy; 2 Italy Campus Bio-Medico University Hospital Foundation, Roma – Italy; 3 Italy University of Rome Tor Vergata, Polyclinic Tor Vergata, Roma – Italy

**Keywords:** Myocarditis, Thymoma, Myocardial Infarction

## Introduction

Thymomas are rare epithelial tumors of the mediastinum frequently associated with paraneoplastic diseases,^1^ myocarditis on the other hand is a rare complication in patients affected by thymoma.^2^ Polymyositis and myocarditis associated with thymoma are exceptionally rare conditions and are usually accompanied by myasthenia gravis (MG).^3^

Myocarditis, an inflammatory disease of cardiac muscle, may result from a wide variety of infectious, toxic, and autoimmune origins. Although the course of the disease is usually self-limiting, acute, non-fulminant myocarditis may progress to fulminant myocarditis. Non-fulminant myocarditis is typically insidious in presentation and may go unnoticed as it progresses into the chronic phase of the disease. The definition of fulminant myocarditis has evolved since its original description in 1991.^4^ In its fulminant form, myocarditis is an illness with hemodynamic derangement and ventricular arrhythmias due to a severe inflammatory process requiring support of cardiac pump function and/or urgent management of serious arrhythmias.^5^ Early recognition and aggressive management are essential for a favorable outcome.^6^ Acute myocarditis may occasionally have an infarct-like presentation, with chest pain, ST‐segment elevation on electrocardiogram, and elevated troponin levels.^7^

We describe a singular case of biventricular fatal fulminant myocarditis with infarct-like presentation occurring in a patient with thymoma.

## Case description

On February 20, a 52-year-old patient was admitted to our Institute with atypical chest pain in the last two weeks.

The patient was a smoker (15 cigarettes a day for about 30 years) and wine drinker (consuming 2-3 glasses of wine a day). Prior right nephrolithiasis. The patient reported a family history of acute myocardial infarction; in their clinical history, relevant diseases were absent.

Thorax RX and thoracic computed tomographic scanning (CT scan) revealed an 11 cm in diameter of anterior mediastinal mass.

At admission the patient had good cardio-circulatory compensation, normal blood pressure (110/70 mmHg), and heart rate of 53 beats/minute; the electrocardiogram (ECG) and the echocardiogram were normal (Figs 1 and 2) in particular, the interventricular septum thickness was normal and there was no evidence of right ventricular wall thickening.

**Figure 1:** – ECG at the admission: Sinus bradycardia, heart rate 53 beats/minute; normal ECG.

**Figure 2:** – Echocardiogram at the admission: the interventricular septum thickness was normal and there was no evidence of right ventricular wall thickening.

A right anterior mediastinotomy, pleurotomy, and mediastinal biopsy were performed.

The biopsy diagnosis was thymoma, probably B2 (according to the WHO classification 2004). A mixture of epithelial cells and lymphocytes was found (Figure 3).

**Figure 3:** – Hematoxylin-Eosin staining (20x) of the mediastinal tumor biopsy: networks and groups of Epithelial cells (black arrows) are seen among T – lymphoid cell infiltration.

Two T cell populations were found by carrying out an immunocytofluorimetry in the peripheral blood and the bone marrow aspirate: however, immunophenotyping showed a reactive polyclonal T-cell lymphocytosis. The hematological consultation, therefore, excluded a clonal T-cell lymphoproliferative disease.

On 14th March 20, the patient underwent neoadjuvant chemotherapy for invasive thymoma: Adriblastina 110 mg and Cisplatin 140 mg. 21 days after chemotherapy, the chest computed tomography showed a reduction of a mediastinal mass.

On 19th May 20, a pain arose in the legs, decreasing with movement; worsening chest pain; and increasing transaminases. The electrocardiogram showed sinus rhythm, heart rate of 89 beats/minute, right bundle branch block, and left anterior fascicular block.

Two days later, due to persistent chest pain and the onset of signs and symptoms of heart failure, an urgent cardiological evaluation was performed and found: undetected blood pressure, undetected carotid pulse, and the electrocardiogram showed wide QRS complex tachycardia, heart rate of 133 beats/minute, right complete bundle branch block, ST segment elevation in V1-V3 simulating an acute myocardial infarction (Figure 4).

**Figure 4:** – ECG 21 May 20: wide QRS complex tachycardia, heart rate 133 beats/minute, right complete bundle branch block, ST segment elevation in V1-V3 simulating an acute myocardial infarction; abnormal ECG.

The echocardiogram showed a moderate reduction in left ventricular ejection fraction (40%), a reduction (qualitative) in right ventricular ejection fraction, dyskinesia of the interventricular septum, an increase in right and left ventricular wall thickness and an increase in interventricular septum thickness (14 mm) (Figure 5).

**Figure 5:** – Echocardiogram 21 May 20: increase in right and left ventricular wall thickness and increase in interventricular septum thickness.

The biochemical markers of myocardium damage were increased: high sensitivity troponin T (hs-cTnT) > 10000 mg/L and creatine-kinase MB mass > 600 mg/ml. Acetylsalicylic acid, clopidogrel, morphine, oxygen, corticosteroids, liquid infusion, and low molecular weight heparin were administered.

### Outcome

Despite the therapy administered, heart failure worsened rapidly and the patient died after a few hours.

## Discussion

At the autopsy, a wide residual necrotic thymoma was found infiltrating the right lung. Autopsy showed biventricular myocarditis resulting in ischemic damage, which was more evident in the right ventricle, with myocardial damage secondary to reactive histiocytic and polyclonal T-lymphoid infiltration (Figure 6).

**Figure 6:** – Myocardium at autopsy: lymphohistiocytic infiltration (black arrows) destroying cardiac muscle fibers (Hematoxylin-Eosin, 10x).

The same type of lymphohistiocytic infiltration was found among the muscle fibers of the peristernal thoracic wall (Figure 7).

**Figure 7:** – Intercostal (anterior thoracic wall) muscles at autopsy: lymphohistiocytic infiltration destroying skeletal muscle fibers (black arrow) (Hematoxylin-Eosin, 20x).

There was secondary reactive biventricular hypertrophy and myocardial and skeletal muscle ischemic damage; the myocardial ischemic damage detected at autopsy could be responsible for the electrocardiographic presentation with ST-segment elevation (myocarditis infarct-like presentation).

The molecular evaluation performed on the biopsy and the autoptic material was negative for the identification of clonal rearrangements of the T cell receptor gamma chain gene.

Myocarditis may be related to several agents/mechanisms,^8^ and among these, to both autoimmune phenomena and drug damage, also by Anthracyclines.

The clinical presentation with symptoms of myocarditis and myositis allowed us to rule out anthracycline-induced myocarditis; in fact, myositis is not described as a complication of anthracycline therapy. Instead, myocarditis and myositis, related to autoimmune phenomena, may be present in patients with thymoma. In addition, the morphological signs of anthracycline-induced myocardial damage are degenerative and necrotic changes in myocytes, vacuolization, mitochondrial damage, and limited focal inflammatory infiltration.^9^ Instead, in our case, a severe and diffuse inflammatory infiltrate was found in the striated muscle cells of the heart and skeletal muscles.

### Final Diagnosis

Diffuse myocarditis resulted in ischemic damage and diffuse myositis (with polyclonal T cell infiltration) in patients with invasive thymoma in treatment. Lung edema and cardiogenic shock.

## Conclusion

Chest pain and dyspnea are frequent symptoms in patients with thymoma, but they may also occur in myocarditis.^8^ Polymyositis and myocarditis associated with thymoma are exceptionally rare conditions and are usually accompanied by MG.

Therefore, in patients with thymoma, it is always necessary to consider the occurrence of myocarditis to establish early appropriate and aggressive therapy: in fact, the inflammation of the myocardium may also develop suddenly and severely, resulting in myocyte necrosis, edema, and cardiogenic shock, specific signs and symptoms of fulminant myocarditis.

Furthermore, the patient with acute myocarditis may present chest pain, ST‐segment elevation on electrocardiogram, and elevated troponin levels. Therefore, a differential diagnosis with acute myocardial infarction must be made.
